# Photobleaching of YOYO-1 in super-resolution single DNA fluorescence imaging

**DOI:** 10.3762/bjnano.8.229

**Published:** 2017-11-02

**Authors:** Joseph R Pyle, Jixin Chen

**Affiliations:** 1 Department of Chemistry and Biochemistry, Nanoscale and Quantum Phenomena Institute, Ohio University, Athens, Ohio 45701, USA

**Keywords:** diffusion, PAINT, single-molecule photophysics, super-resolution imaging

## Abstract

Super-resolution imaging of single DNA molecules via point accumulation for imaging in nanoscale topography (PAINT) has great potential to visualize fine DNA structures with nanometer resolution. In a typical PAINT video acquisition, dye molecules (YOYO-1) in solution sparsely bind to the target surfaces (DNA) whose locations can be mathematically determined by fitting their fluorescent point spread function. Many YOYO-1 molecules intercalate into DNA and remain there during imaging, and most of them have to be temporarily or permanently fluorescently bleached, often stochastically, to allow for the visualization of a few fluorescent events per DNA per frame of the video. Thus, controlling the fluorescence on–off rate is important in PAINT. In this paper, we study the photobleaching of YOYO-1 and its correlation with the quality of the PAINT images. At a low excitation laser power density, the photobleaching of YOYO-1 is too slow and a minimum required power density was identified, which can be theoretically predicted with the proposed method in this report.

## Introduction

Fluorescence imaging of DNA with intercalating dyes is important for DNA sensing [1–2], nucleic acid imaging inside cells and viruses [3–5], DNA protein studies [6–7], and optical mapping [8–10]. YOYO-1 is a common dye chosen for these studies due to its favorable optical properties. YOYO-1 has a high extinction coefficient of 10^5^ M^−1^ cm^−1^ [11] and strongly binds to DNA (binding constant 10^8^–10^9^ M^−1^) [12] with little sequence preference. Its fluorescent brightness at visible wavelengths is enhanced over 1,000-fold upon intercalation into DNA as compared to free YOYO-1 in water [13–15], which has triggered a revolution in DNA labeling since the 1990s [16].

**Figure 1:**
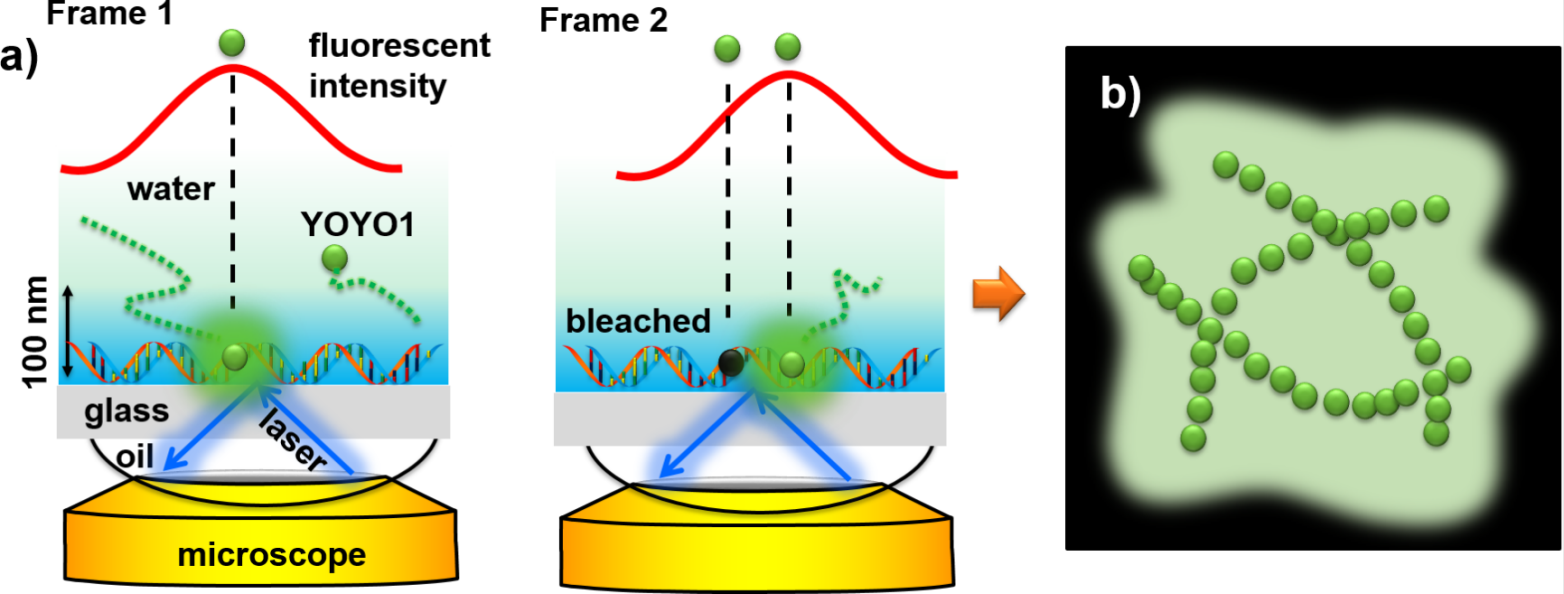
Scheme of super-resolution imaging of DNA with PAINT. (a) A scheme depicting the localization of single molecules in each frame. (b) Scheme of the reconstruction of a super-resolution image on top of a regular fluorescence image from the super-localized molecules in each frame.

YOYO-1 has been one of the major dyes used for super-resolution DNA imaging [17–20]. A recent trend in fluorescent imaging is the use of super-resolution imaging to resolve fine structures below the typical diffraction limit of visible light microscopy at ≈250 nm [21]. This is important in visualizing the conformation of DNA molecules, such as DNA looping by proteins, a necessary process for gene regulation and expression [22], characterizing DNA origami [23–24], and imaging the unpacking of DNA [25]. Two main categories of super-resolution techniques were developed in the past two decades: (1) using hardware to beat the diffraction limit, using methods such as stimulated emission depletion (STED) microscopy [26–27]; (2) using software to super-localize single molecules [28–31], such as stochastic optical reconstruction microscopy (STORM) [32], photo-activated localization microscopy (PALM) [33], single-molecule high-resolution imaging with photobleaching (SHRIMP) [34], and point accumulation for imaging in nanoscale topography (PAINT) [35–36]. The main principle behind the latter techniques is to take a fluorescent video of single molecules over time. Each frame of the video is then processed to determine the center of each fluorescent point spread function (PSF) by fitting it to, for example, a Gaussian function (Figure 1). Then all the frames are overlaid to construct the super-resolved image. The difference between each technique is how the single molecules are visualized, typically through blinking, photobleaching, binding activation, photoswitching, or a combination thereof [34,37–38]. Dye photobleaching is one of the most utilized methods in PAINT fluorescently turn-off the dye molecules and is commonly used in most all types of fluorescent imaging [17,19–20,39–40]. Thus, carefully tuning the photobleaching rate is an important step for super-resolution imaging. However, finding suitable photobleaching lifetimes for YOYO-1 in PAINT imaging has not been reported in the literature.

In this paper, we study the effect of laser power on both conventional fluorescent imaging and PAINT imaging of single DNA molecules using the intercalating dye YOYO-1. YOYO-1 is very dim in aqueous solutions and is bright when intercalated into the DNA molecule. Thus, stochastic binding and photobleaching of YOYO-1 molecules enable PAINT imaging (Figure 1). While high laser power is desired for higher resolution and fast photobleaching of the bound YOYO-1, low laser power is also desired to reduce photodamage to the immobilized DNA and to the YOYO-1 molecules in the bulk solution [39,41]. Thus, the effect of laser power is an important parameter to control during imaging to maintain single-molecule fluorescence while also preserving the DNA from photocleavage (photodamage).

## Experimental

### Sample preparation

All λ-DNA (Thermo Fisher) and YOYO-1 (Invitrogen) solutions were prepared in the buffer of 10 mM HEPES (pH 7.4, Acros Organics) with 10 mM NaCl (Sigma-Aldrich). All water used was from a Barnstead E-Pure ultrapure water purification system with a resistivity of 18 MΩ cm^−1^.

Glass coverslips were first cleaned by sonication in 1% detergent (Liquinox) followed by rinsing with 18 MΩ water. Then the coverslips were immersed in 1:1:5 (v/v/v) of ammonium hydroxide/hydrogen peroxide/water for 15 min at 60 °C. Afterwards they were rinsed with water and dried with nitrogen. The coverslips were immersed in a solution of 1 vol % 3-aminopropyltriethoxysilane (APTES, TCI America) in HPLC grade acetone (Fisher) at 50 °C for 20 min. Afterwards, they were washed with ethanol and water and dried under nitrogen.

PDMS blocks were made by thoroughly mixing Sylgard 184 silicone elastomer base (Dow Corning) with the provided curing agent (10:1 by mass), which was poured into a Petri dish, vacuum desiccated to remove bubbles, and cured overnight. After curing, the PDMS blocks were cut into similar sizes as the coverslips. Syringe tips were inserted through the PDMS block. These tips were cut and connected to tubes. Microfluidic channels were constructed by adhering double-sided tape to the coverslip and the PDMS. A rectangle was cut out of the tape before adhering to the coverslip to form a channel with dimensions of approximately 2 cm × 2 mm × 30 µm (length, width, height). The height is defined by the thickness of the tape.

### Fluorescence imaging

**Figure 2:**
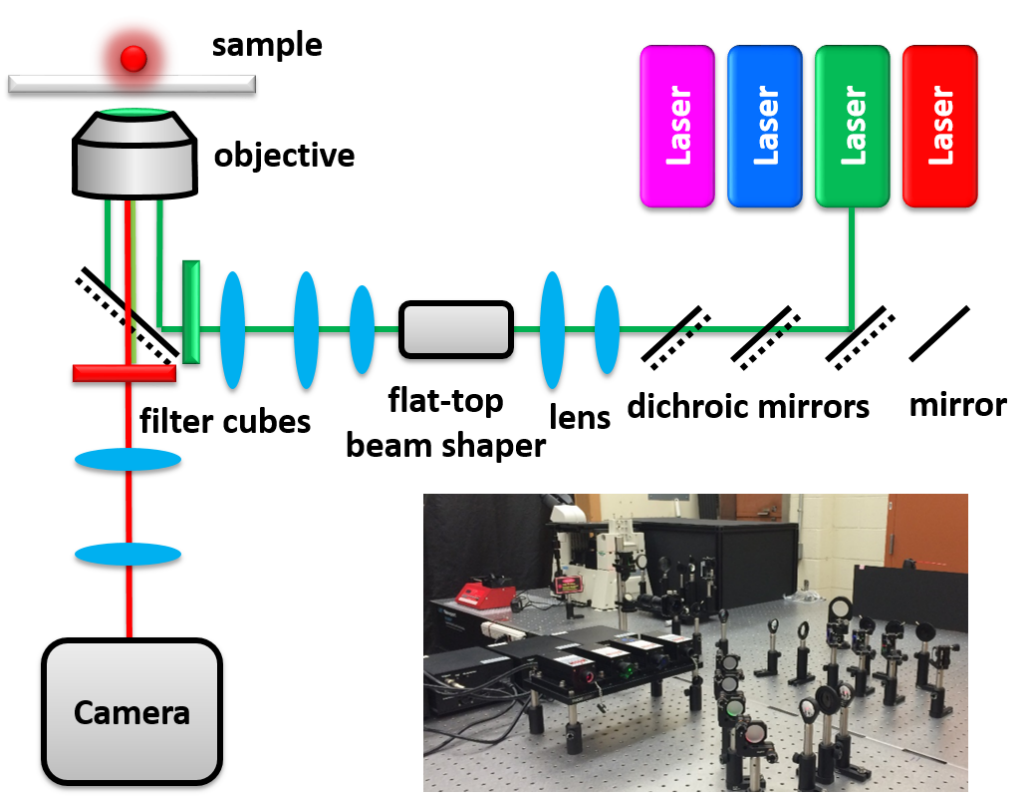
Scheme and image of the optical microscope.

All fluorescence measurements were carried out with a home-built microscope under total internal reflection fluorescence (TIRF) mode (Figure 2) equipped with four solid state lasers (Dragon Lasers, China), two beam expanders and a flat-top beam shaper (piShaper, AdlOptica GmbH, Germany), in addition to a Nikon Ti-U inverted microscope with a Nikon 100× oil-immersed TIRF objective (CFI Apo 100×, NA 1.49, WD 0.12 mm), and an EMCCD camera (Andor iXon Ultra 897). TIRF fluorescent filter cubes were equipped with a microscope for each laser source. The blue fluorescent filter cube used in this study was a model TRF49904 (Chroma) for the 473 nm laser.

All diffraction-limited experiments were accomplished using 1:10 dye/base pair YOYO-1-stained λ-DNA. The YOYO–DNA was incubated at 50 °C for two hours to achieve homogeneous staining as described by Carlsson et al. [42]. Approximately 200 µL of the YOYO–DNA solution was injected into the microfluidic channel using a syringe pump (New Era Pump Systems Inc., model NE-1000) at 0.40 mL/min. YOYO–DNA adhered to the surface of the amine-modified glass through electrostatic attraction between the negatively charged phosphate groups in the DNA backbone and the positively charged amine groups on the surface. The DNA molecules were stretched by flow through the channel. The channel was then washed with buffer solution to remove the non-immobilized YOYO–DNA.

### Single DNA intensity measurements

Short videos of single YOYO–DNA molecules (≈200 frames) using 50 ms integration time and an electron-multiplying (EM) gain of 200 were obtained. To limit the effect of bleaching on the measurements, the sample was focused under illumination of a low-power 532 nm laser. Then the 532 nm laser was blocked and the video started recording in the dark. Then the 473 nm laser with a measured power was switched on. A MATLAB code was used to select the area of single YOYO–DNA molecules. The frame when the DNA first appeared was designated as time zero.

### Bleaching lifetime measurements

Identical experimental conditions were used as described above except much longer videos were taken (1000–2500 frames) in order to monitor longer YOYO-1 bleaching. The intensity of each YOYO–DNA molecule was found in each frame. A double exponential function was used to fit the decay data using a home-written MATLAB code.

### Super-resolution imaging

The microfluidic channels (described earlier) were washed with water and then ≈0.1 mL of 300 pg/µL λ-DNA in the buffer was flowed through at a rate of 0.4 mL/min followed by 1 mL of buffer solution to wash away any excess DNA that did not bind to the surface. YOYO-1 (5 nM) was flowed through the channel at 0.05 mL/min while recording 5100 frame videos of binding to DNA under TIRF illumination from the 473 nm laser with an exposure time of 50 ms and an EM gain of 200. The super-resolution images were constructed using a MATLAB code that has been previously described [43–45].

## Results and Discussion

### Conventional DNA immobilization and imaging

All fluorescence measurements were carried out on a home-built optical microscope (Figure 2). The power density of the illumination from the 473 nm laser is calculated from the total illumination power over the illumination spot area. This calculation is reasonable because our flat-top beam shaper tunes the power density within the illumination spot uniformly. Without the beam shaper, the illumination intensities in the spot are usually Gaussian distributed. The total power is measured using a light detector (Op-2-Vis, Coherent) after the laser passes through a control sample that has no absorbers on it under epifluorescence mode. The spot size of the 100× (1.49 NA) objective was determined by bleaching polymer dots (PF-TC6FQ-Pc) [46] and then viewing the bleached area in the 20× objective (0.50 NA). The bleaching profile (Supporting Information File 1, Figure S1) is uniform in the beam spot indicating a relatively uniform light distribution of the laser after the flat-top beam shaper, consistent with a relatively even photocount distribution in a typical fluorescent image. Note that the actual photons emitted from a molecule is a function of the measured photocounts, the EM gain (fixed at 200× during all measurements), and the photon collection efficiency of the optical pathway [47–48]. The number of photocounts is at the linear response region of the EMCCD under our imaging conditions [48].

**Figure 3:**
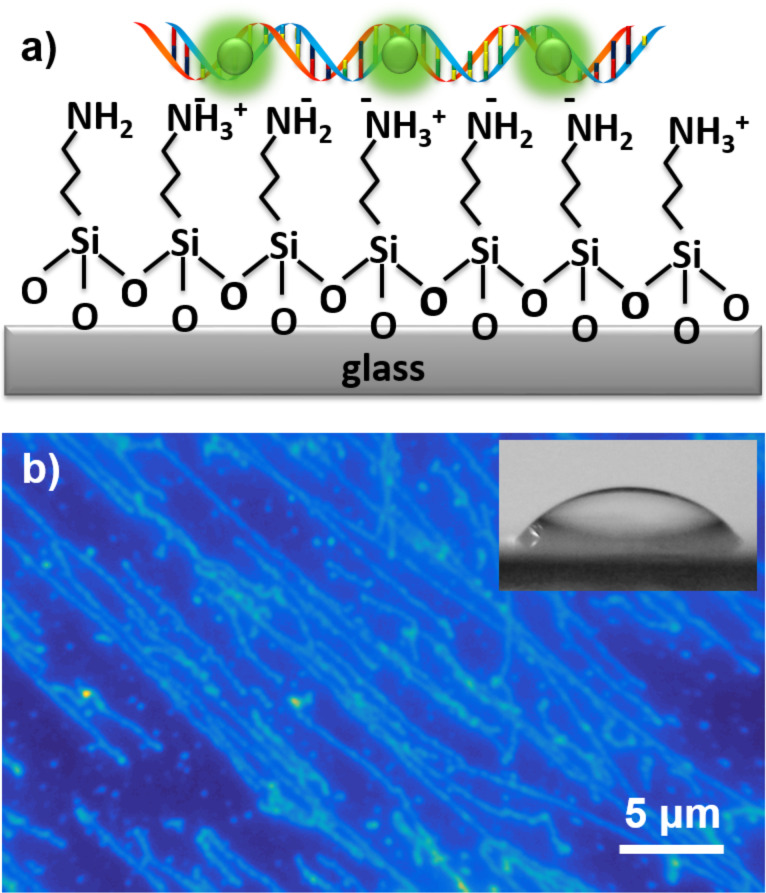
(a) Scheme of glass surface modification and DNA immobilization. (b) Fluorescent image of stretched DNA molecules on the substrate that have been labeled with YOYO-1. The inset shows a droplet of water on the substrate before DNA immobilization, giving a water contact angle of 48 ± 4°.

We can stretch, immobilize, and image a single DNA using the established protocol and our fluorescence microscope. Glass cover slip substrates modified with amino silane are used to immobilize single DNA molecules (Figure 3). After surface modification, the water contact angle for the cover slips is 48 ± 4° which is consistent with the literature for such surfaces [49–51]. These modified cover slips are then used to fabricate flow channels for DNA imaging. YOYO-1 is mixed and incubated for 2 h at 50 °C with a λ-DNA solution before immobilization. The DNA solution is then flowed into the channel with a flow speed (0.4 mL/min) that is capable of stretching the DNA molecules. The DNA is negatively charged and the surface is positively charged in the buffer solution. Thus, the electrostatic interaction between them provides the immobilization force for the DNA molecules. The stretched DNA molecules are visualized under the microscope showing that sample preparation and our microscope work performed as expected (Figure 3b).

**Figure 4:**
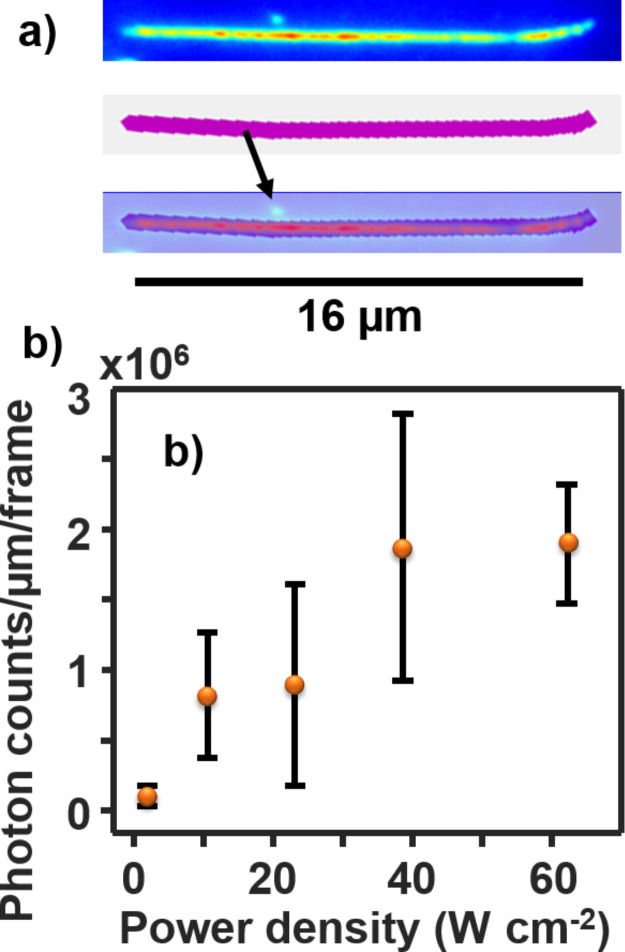
(a) A single DNA molecule (top), the mask used (middle), and overlay (bottom) to select the DNA and filter the other events (the arrow). (b) The intensity (total photocounts) per micrometer of single DNA molecules as a function of laser power density. The images are obtained under TIRF mode but the power densities are measured under epifluorescence mode. YOYO-1 molecules are premixed with λ-DNA at a dye/base pair ratio of 1:10. The error bars represent the standard deviation between at least 25 DNA for two samples.

In order to measure the heterogeneity among DNA molecules, DNA molecules were immobilized on the substrate at a lower density than in Figure 3. This lower density allows the DNA molecules to be separated from each other. They are manually removed from the fluorescent image using in-house developed MATLAB code to avoid errors from other events (Figure 4a). Then the single DNA fluorescence intensity is calculated as the total photocounts per micrometer (μm) length of the λ-DNA molecules (Figure 4b). Only well-separated single DNA molecules are chosen for the purpose of easy quantification. The DNA strands are prelabeled with YOYO-1 at a dye–DNA base pair ratio of 1:10. The average dye concentration in each DNA is calculated to be ≈300 dye molecules/μm for a given length of a DNA molecule, assuming 0.34 nm per base pair and that the DNA is fully stretched.

A linear trend is observed which is expected at laser powers low enough for single photon absorption. The two-photon absorption probability can be estimated by comparing the number of photons absorbed by the dye per unit time to the fluorescence lifetime of the dye. The number of photons absorbed can be estimated using the following equation [47]: Photon absorption = σ*P*/*E*_ph_, where σ is the absorption cross section, *P* is the laser power density and *E*_ph_ is the energy of a photon. The photons absorbed by each YOYO-1 molecule at the highest laser power studied (62 W cm^−2^) is calculated to be 24 photons/ms using the energy of a photon at 473 nm (4.2 × 10^−19^ J/photon) and calculating the absorption cross section, 1.64 × 10^−16^ cm^2^ that is calculated from the extinction coefficient, 9.89 × 10^7^ cm^2^ mol^−1^ (≈10^5^ M^−1^ cm^−1^) [11]. Since the fluorescence lifetime of YOYO-1 in DNA is 2–5 ns [52], that is, much shorter than the photon flux interval, all laser powers studied in this work should not be high enough for two-photon absorption to occur. Thus, the error in the experiment is due to both inhomogeneous dye staining and DNA stretching quality (Supporting Information File 1, Figure S2).

### YOYO-1 photobleaching in a single DNA

**Figure 5:**
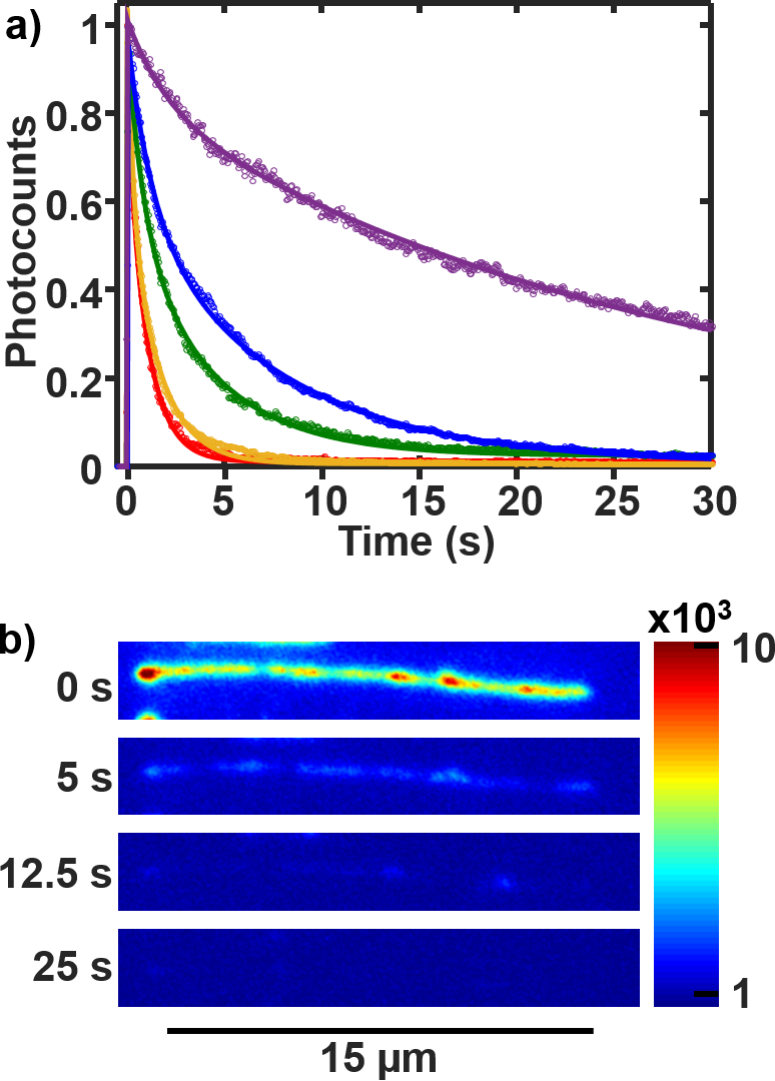
(a) The time trace of the photocounts per micrometer of a single DNA strand for power densities of 62 W cm^−2^ (red), 38 W cm^−2^ (orange), 23 W cm^−2^ (green), 10 W cm^−2^ (blue), and 1.9 W cm^−2^ (violet). (b) Image of a DNA molecule at different times of the video. All images have the same color scale. A laser power density of 38 W cm^−2^ was used.

Photobleaching lifetimes of the YOYO–DNA were measured from the video of YOYO-1 labeled and immobilized DNA. PAINT imaging of DNA requires bleaching of YOYO-1 to maintain single-molecule fluorescence (the sparsity principle). YOYO-1 binds very strongly to DNA (*K*_a_ = 10^8^–10^9^ M^−1^) [12], and stays in the DNA for a long time. If not bleached, the whole DNA strand will eventually light up instead of a few isolated dye molecules per frame. Thus, the bleaching lifetime of YOYO-1 should be tuned for PAINT. The bleaching lifetime can be obtained from the fluorescent intensity decay of the molecules in the video (Figure 5). From the bulk experiments, YOYO-1 photobleaching kinetics can be fitted with a double-exponential decay function to represent the photobleaching of two differently bound YOYO-1 molecules: intercalated and non-intercalated [40]:

[1]
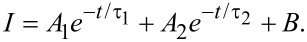


**Figure 6:**
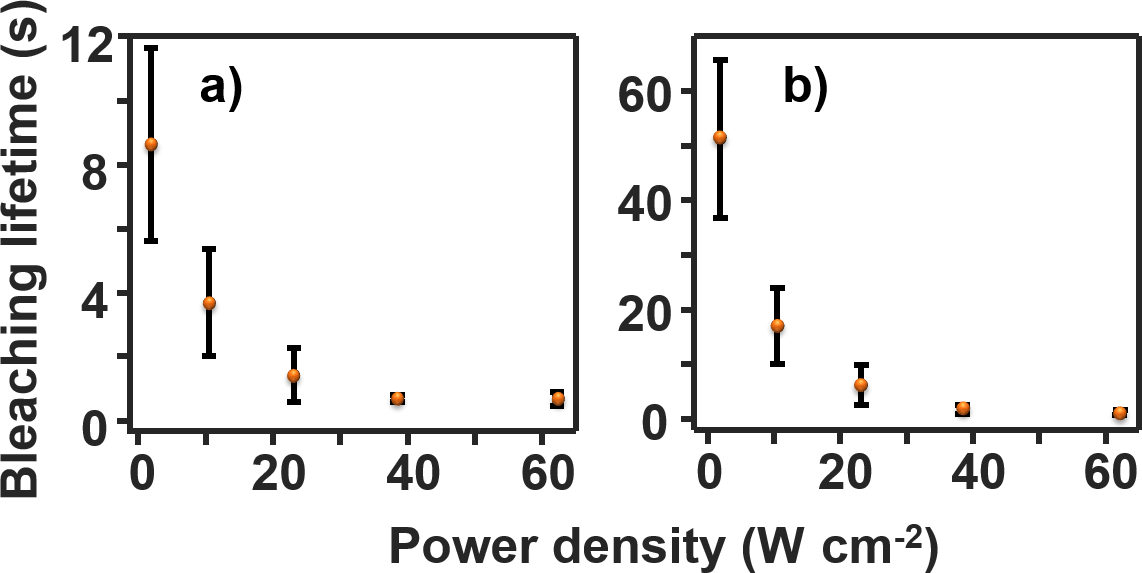
(a) The fast component of the bleaching lifetime for YOYO–DNA as a function of power density. (b) The slow component of the bleaching lifetime for YOYO–DNA as a function of power density. The error bars represent the standard deviation between at least 20 DNA for two samples.

The average bleaching lifetime shows an exponential-like decay with increasing laser power (Table 1, Figure 6). For the two highest powers, 38 and 62 W cm^−2^, the fast lifetime is ≈1 s. Thus, ≈3% of molecules are bleached within the first imaging frame, 50 ms, and ≈2/3 are bleached within the first second of laser exposure.

**Table 1:** Average fitting constants for the double-exponential bleaching curves (error bars are shown in Figure 6).

Power density (W cm^−2^)	*A*_1_	τ_1_ (s)	*A*_2_	τ_2_ (s)	*R*^2^

62	2 × 10^6^	0.68	1.1 × 10^6^	1.2	0.97
38	3 × 10^6^	0.69	1.7 × 10^6^	1.7	0.98
23	9 × 10^5^	1.4	5 × 10^5^	6.3	0.98
10	7 × 10^5^	3.7	2 × 10^5^	17	0.99
1.9	4 × 10^4^	8.6	5 × 10^4^	51	0.99

### Super-resolution imaging

A super-resolution image of DNA molecules is obtained from analyzing each video when non-labeled DNA is immobilized and YOYO-1 (5 nM) is flowed in for in situ labeling. The theoretical resolution of a PAINT image is dependent on the photocounts per molecule during the imaging period. The theoretical square uncertainty of a super-resolved dye location using Gaussian fitting of the point spread function of a dye can be calculated with the Thompson equation [28,53]:

[2]
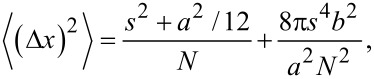


where *s* is the standard deviation of the point spread function, *a* is the size of a pixel, *N* is the number of photons collected, and *b* is the background noise.

**Figure 7:**
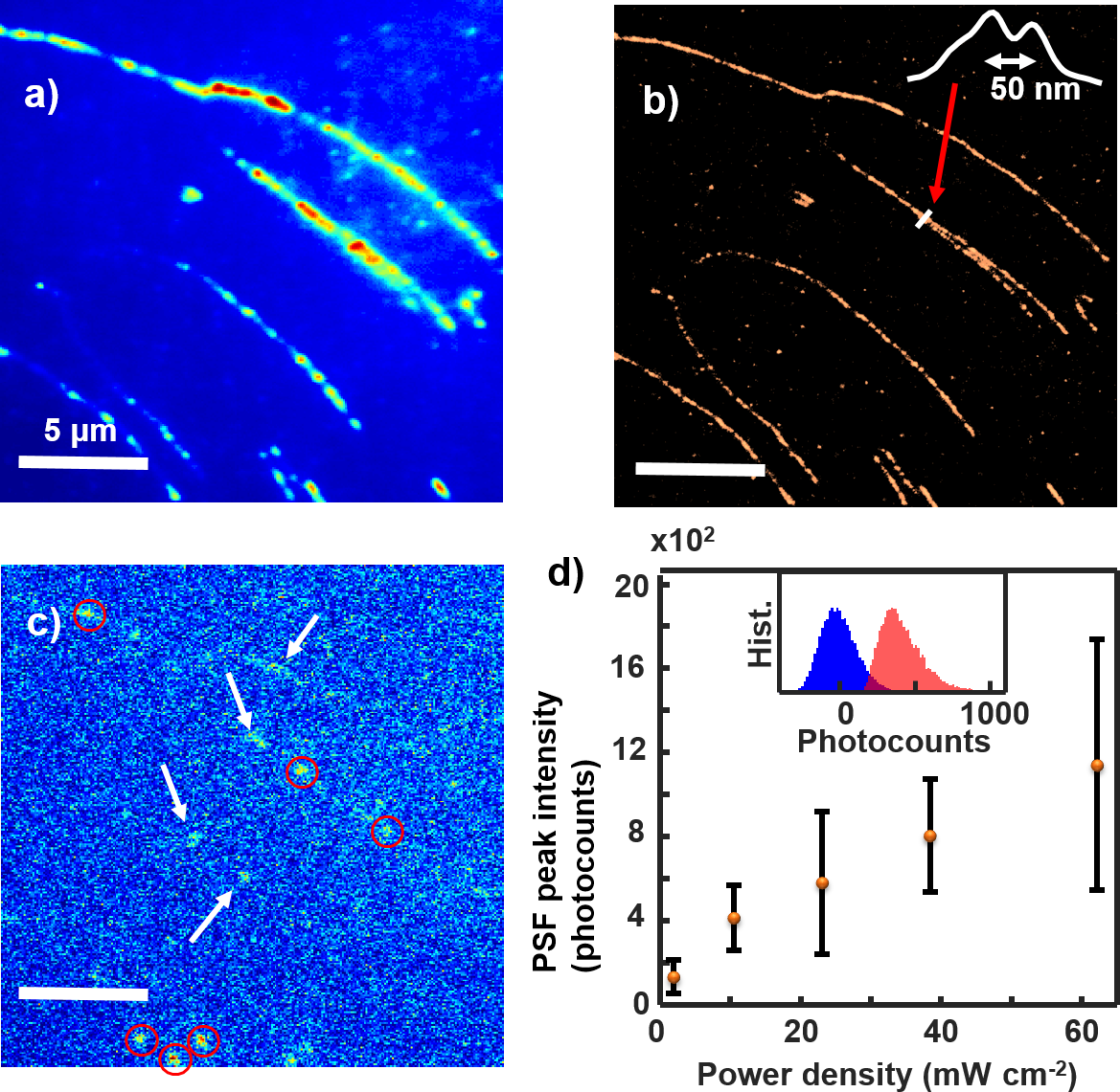
(a) Fluorescence images of regular and (b) super-resolved YOYO–DNA (inset shows a line profile across two nearby DNA molecules). Laser power density 23 W cm^−2^. (c) A single frame of the video of fluorescent images. The circles are events chosen and the arrows are events discarded in generating the image in (b). (d) PSF peak intensity as a function of laser power density (inset shows histograms of the background and PSF peak intensities, see Supporting Information File 1, Figure S4 for larger images). The error bars represent the standard deviation of the photocounts of YOYO-1 molecules for three samples.

The resolution of the experimental measurements (PAINT) is consistent with the theoretical prediction. Figure 7 shows the regular fluorescent image (Figure 7a) and the super-resolved image (Figure 7b) of YOYO–DNA at a power density of 23 W cm^−2^. In Figure 7, on average, *s* = 112 nm, *a* = 72 nm, and *b* = 120 counts. The total photocounts of a dye can be calculated by integrating the fitted volume under each point spread function (PSF) *N* = 2π *A*_PSF_ σ*_x_* σ*_y_*, where *A*_PSF_ is the PSF peak intensity in photocounts, and σ*_x_* and σ*_y_* are the fitted Gaussian standard deviation in pixel units (Supporting Information File 1, Figure S3). In Figure 7, *A*_PSF_ = 420 counts, and σ*_x_* = σ*_y_* = 1.5 pixels, so *N* ≈ 6000 counts. This value is consistent with the sum of the photocounts of all pixels in a measured PSF, which is proportional to the actual photon emission at the linear detector response region [47–48]. Thus, the theoretical uncertainty is ≈17 nm. The full width at half maximum (FWHM) will be ≈40 nm if multiple events of each dye are represented in a Gaussian distribution. This value is consistent with our experimental measurement of ≈50 nm shown in Figure 7b. The resolution of a regular fluorescent image is ≈300 nm corresponding to the FWHM of the PSF of a single dye. Thus, when two DNA are close, conventional fluorescent imaging cannot resolve them. The super-resolved image has ≈50 nm resolution and can resolve DNA molecules greater than this separation. The contrast of the super-resolution image is also better than the regular fluorescent image because some background has been filtered out and the weight of the very bright dye molecules has been reduced. Figure 7c shows a single frame from the 5000-frame video involved in generating Figure 7b. The frames contain two signals, background and single-molecule fluorescent emission. The background is represented by a Gaussian distribution whose center is set to zero photocounts. The fluorescent molecules are identified when its maximum has intensity larger than three times the standard deviation of the background distribution. Because the background varies over frames and regions on the images, a local background method is used instead of the global background [45]. The distributions of the background and the PSF maximum are shown in Figure 7d, where the average PSF maximum is ≈3.5 times the standard deviation of the overall background distribution. Several bright spots of fluorescent dye molecules (events) are identified on the frame in Figure 7c. They are separated into two groups, a group that is consistent with the single-molecule PSF (circles) and the other group (arrows) that is not because of irregular shapes and/or too large sizes. The former locations are stored and the latter locations are filtered out. The PSF peak intensities of single YOYO-1 dyes are shown in Figure 7d. They are calculated from the center of the peak intensity histograms of single YOYO-1 molecules (Supporting Information File 1, Figure S3). The graph is roughly linear which is expected at this range of power densities.

**Figure 8:**
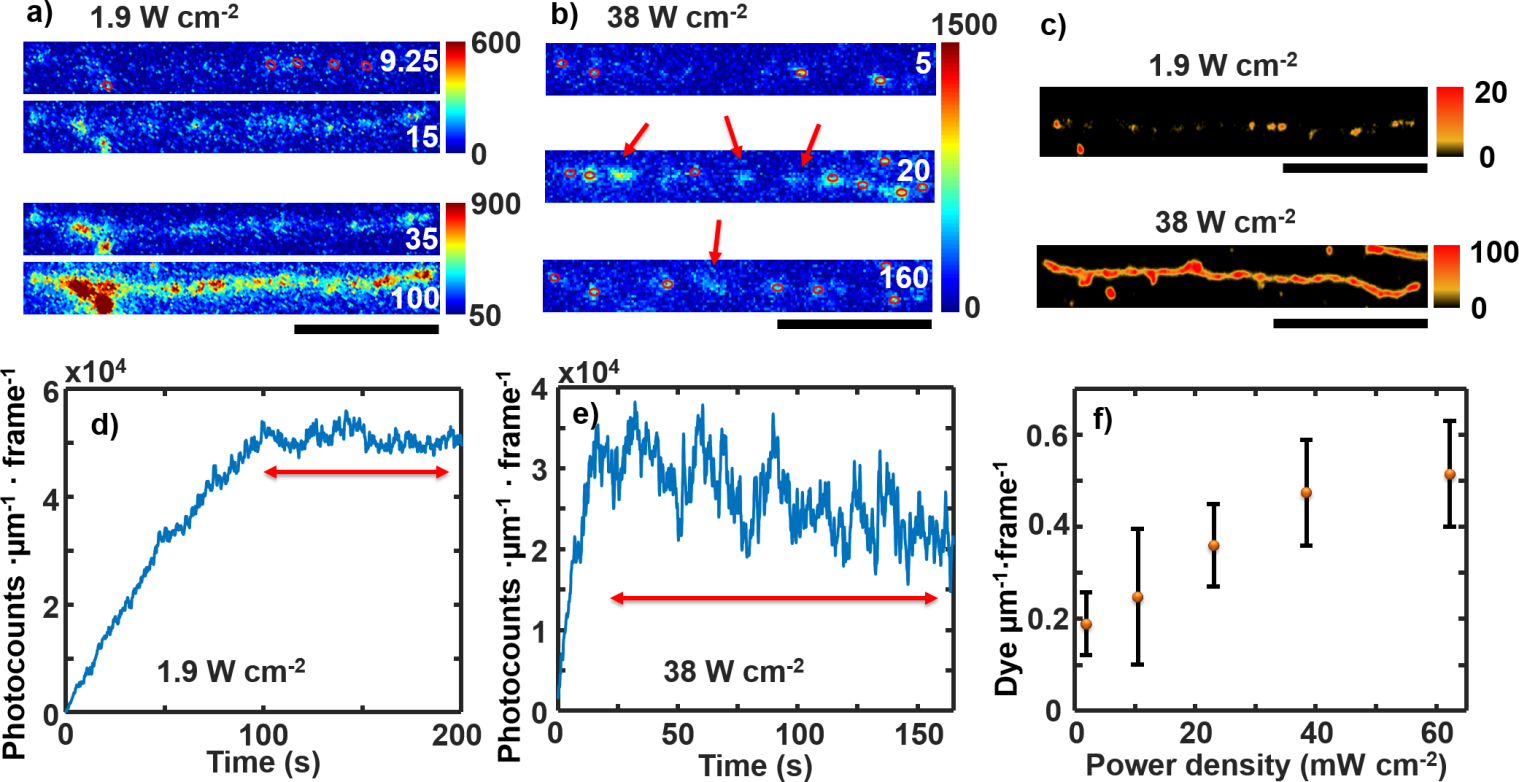
Single-frame images of a single DNA molecule at (a) 1.9 W cm^−2^ and (b) 38 W cm^−2^ power densities (time shown is in unit of seconds) and (c) corresponding PAINT images. Red circles show events selected and arrows show the events discarded by the code and bleached later. All scale bars are 5 μm. (d, e) Normalized total photocounts of 1 μm of DNA in each frame at 1.9 W cm^−2^ and 38 W cm^−2^ power densities, respectively. The red double arrows indicate where equilibrium is maintained. (f) The average number of dye molecules selected in the frames during the PAINT analysis. The error bars represent the standard deviation between ≈20 DNA for two samples.

Resolving DNA strands with PAINT requires an optimal power density to view single dye molecules throughout the video (Figure 7c, Figure 8). During a PAINT image acquisition, YOYO-1 molecules continuously bind to the DNA and eventually fill the whole DNA strand. At a certain laser power photobleaching prevents the YOYO-1 from being observed and establishes an equilibrium for single-molecule imaging. Figure 8a,b shows selected frames from two example videos of YOYO-1 binding to non-labeled DNA molecules. At a low power density, 1.9 W cm^−2^, almost the entire strand is visible at ≈35 s (Figure 8a). This limits PAINT from identifying single dyes (against the sparsity principle). Thus, the useful time range for PAINT is the first 15 s of data acquisition even though the equilibrium between the binding and the bleaching is reached at a time after 100 s (Figure 8d). This short time is not enough to generate a complete super-resolution image of the DNA stands (Figure 8c). At a higher laser power density, 38 W cm^−2^, YOYO-1 molecules continuously bind at the same rate but are bleached at a higher rate (Figure 6). Thus, the equilibrium is reached faster at ≈20 s (Figure 8e). Because single-molecule separation is still clearly seen at equilibrium (Figure 8b), the whole video can be used to generate the PAINT image (Figure 8c) and the video can run even longer until the DNA is saturated with YOYO-1.

**Figure 9:**
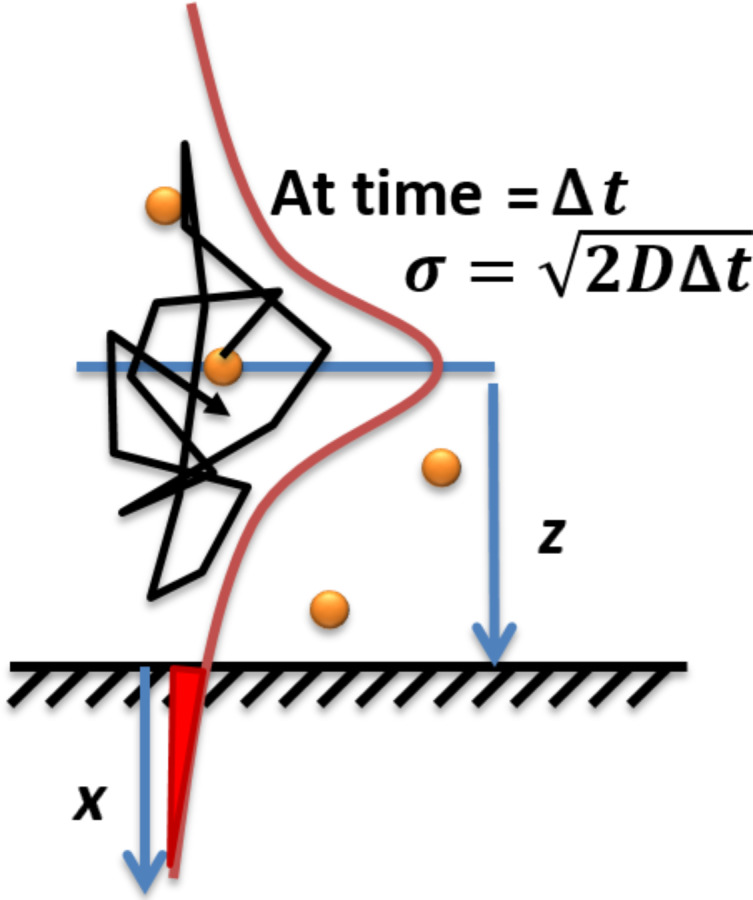
Scheme of molecular diffusion in the solution.

The theoretical YOYO-1 binding rate can be estimated with Einstein’s Brownian motion and Fick’s second law: the YOYO-1 molecules diffuse in the solution where the location probability is a Gaussian distribution after an evolution time Δ*t* (Figure 9),

[3]
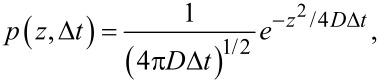


where *p*(*z*, Δ*t*) is the probability distribution of a diffuser in the solution at time Δ*t* over one dimension *z*, and *D* is the diffusion constant. The integration of the error function of this distribution over all molecules in the solution represents the hitting rate (HR) of the molecules to a substrate (Figure 9):

[4]
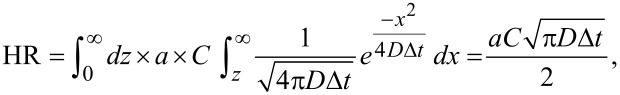


where *a* is the surface area, *C* is the concentration, and Δ*t* is the evolution time (frame time here). The diffusion constant can be estimated using the Stokes–Einstein equation assuming no frequency dependence at our measuring window:

[5]
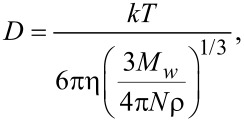


where *k* is the Boltzmann constant, *T* is the temperature, η is the solution viscosity, *M_w_* is the solute’s molecular weight, *N* is Avogadro’s number, and ρ is the solute solid density. The molecular weight of YOYO-1 is 1271 g mol^−1^, its density can be estimated to be 0.8 g cm^−3^, and the viscosity of water is 8.9 × 10^−4^ Pa s. At room temperature, the diffusion constant of YOYO-1 in water is ≈2.9 × 10^−10^ m^2^ s^−1^. The area of 1 μm of double-stranded DNA is ≈2 × 10^−15^ m^2^. The YOYO-1 concentration is 5 nM, and the frame time is 50 ms. Thus, every frame has 0.02 YOYO-1 molecules that hit every 1 μm length of a DNA molecule (0.4 s^−1^). This value is consistent with our measurement (Figure 8c). The slope of the 1.9 W cm^−2^ curve at the first 10 s is ≈600 counts/s when photobleaching is insignificant. The PSF peak intensity at this laser power is ≈100 counts, representing a total photocount per molecule of ≈1400. Thus, an increase of ≈0.4 molecules per second is observed in this increasing part of the 1.9 W cm^−2^ slope which is consistent with the theoretical calculation. Note that this is only tested at the fixed flow rate and frame rate. This agreement suggests that under this condition, the electrostatic interaction between YOYO-1 and DNA does not provide an effective area for the YOYO-1 to bind that is larger than its physical size. This is reasonable because the Debye length of our buffer solution is ≈2 nm, while YOYO-1 molecules are separated from each other by ≈700 nm (5 nM), where the long range interaction is negligible. It also indicates an efficient binding of YOYO-1 onto DNA that is consistent with the large binding constant measured in ensemble (i.e., every YOYO-1 molecule interacting with DNA gets caught).

The competition between binding and bleaching regulates the number of useful video frames for PAINT imaging. Figure 8f shows the average dyes selected by PAINT in the frames at different power densities. The average useful dye molecules increase with increasing power density. A preliminary analysis shows that the balance between binding and bleaching is reached around a power density of 23 W cm^−2^. Below this power density, the bleaching is too slow to remove adjacent dyes, and above this power density, bleaching is fast enough to satisfy single-molecule imaging. At 23 W cm^−2^, ≈0.35 dye molecules are observed per 1 μm length of DNA per frame (one dye molecule every ≈3 µm of DNA). Less than 10 dye molecules (≈3/0.3) can be filled in this length on average to maintain the single-molecule separation (PSF overlap avoided), where 0.3 µm is the FWHM of the PSF. Assuming a new dye molecule arrives randomly to an area with one existing dye molecule, there is a 3/10 probability that a new one will hit the area on top or nearby this existing dye molecule. Roughly three dye molecule hitting cycles are left, which takes ≈3/0.02 = 150 frames to reach without photobleaching, where 0.02 dye molecules per frame per 1 μm length of DNA is the average binding rate we have measured. Thus, the existing dye molecule must be bleached within ≈150 × 0.05 s = 4.5 s. This value is consistent with the average photobleaching lifetime measured at this power density, that is ≈3 s (Table 1). The same calculations for lower power density yield a required photobleaching lifetime that is shorter than that measured, thus only the beginning of the videos are useful. However, for higher power densities, the measured bleaching lifetimes meet the requirement. This is confirmed by the visual analysis of single frames at the equilibrium stage (Figure 8a,b).

Thus, the required power density is predictable. In order to obtain the whole DNA image at a resolution of 50 nm, ≈20 total dye molecules per micrometer of DNA are required, which requires 20/0.02 = 1000 frames or 50 s for the YOYO-1 molecules to diffuse to the DNA under the current YOYO-1 concentration (5 nM). If 10 s is desired to image the whole DNA, a 25 nM YOYO-1 concentration should be used instead. Under this concentration, YOYO-1 binds to DNA at an average of 0.1 per frame per 1 μm length of DNA, which requires a photobleaching lifetime of each YOYO-1 molecule to be ≈30 × 0.05 s = 1.5 s in order to resolve single YOYO-1 molecules at the frame time of 50 ms per frame. This YOYO-1 photobleaching lifetime requires ≈50 W/cm^2^ laser power density under our experimental conditions (Figure 6). Lower laser power densities can be used with the help of data analysis methods that tolerate a higher active-dye density and slight overlap of the PSFs, such as SHRIMP [34,54], Bayesian analysis of the blinking and bleaching (3B) [37,55], compressed sensing [56], and super-resolution optical fluctuation imaging (SOFI) [57–58], or methods that provide higher time resolution such as supertemporal-resolved microscopy (STReM) [59].

## Conclusion

We have measured the single-molecule photobleaching lifetimes of YOYO-1 dye in DNA at different excitation laser power densities. We have also established a correlation between the photobleaching lifetimes with the quality of the super-resolution PAINT images. Under PAINT conditions, the dye molecules in the solution continuously bind to the target surfaces and are photobleached by the excitation laser. In order to maintain single-molecule resolution (the sparsity principle), they have to be photobleached fast enough, using a power density as low as possible to avoid photodamage to the samples. In this work, we are able to screen a set of power densities to find this optimal value of the power density and a generalized method is provided to estimate it theoretically.

## Supporting Information

Additional experimental information.

File 1 Laser spot size, DNA length, PSF, and photocount histograms.
