# Correction: Photobleaching of YOYO-1 in super-resolution single DNA fluorescence imaging

**DOI:** 10.3762/bjnano.9.74

**Published:** 2018-03-06

**Authors:** Joseph R Pyle, Jixin Chen

**Affiliations:** 1 Department of Chemistry and Biochemistry, Nanoscale and Quantum Phenomena Institute, Ohio University, Athens, Ohio 45701, USA

**Keywords:** diffusion, PAINT, single-molecule photophysics, super-resolution imaging

The originally published Figure 7 and Figure 8 contain mistakes.

In Figure 7d and in Figure 8f the *x*-axis labeled unit should be W cm^−2^ instead of mW cm^−2^. Figure 1 in this Correction shows the corrected Figure 7 of the original publication and Figure 2 shows the corrected Figure 8 of the original publication.

On page 2304, left column line 13 from the bottom 4.5 s should be 7.5 s.

**Figure 1:**
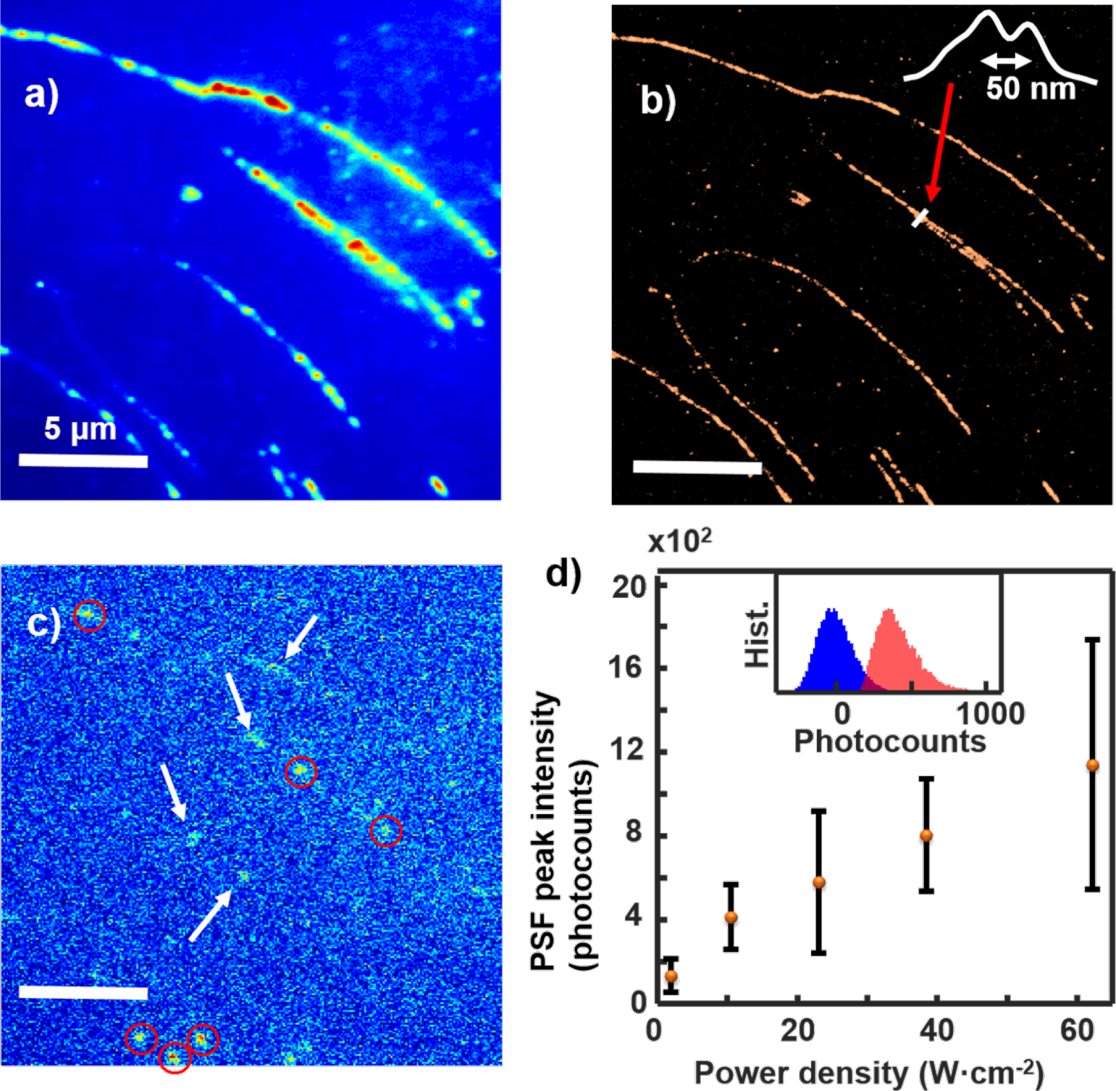
Corrected Figure 7 of the original publication.

**Figure 2:**
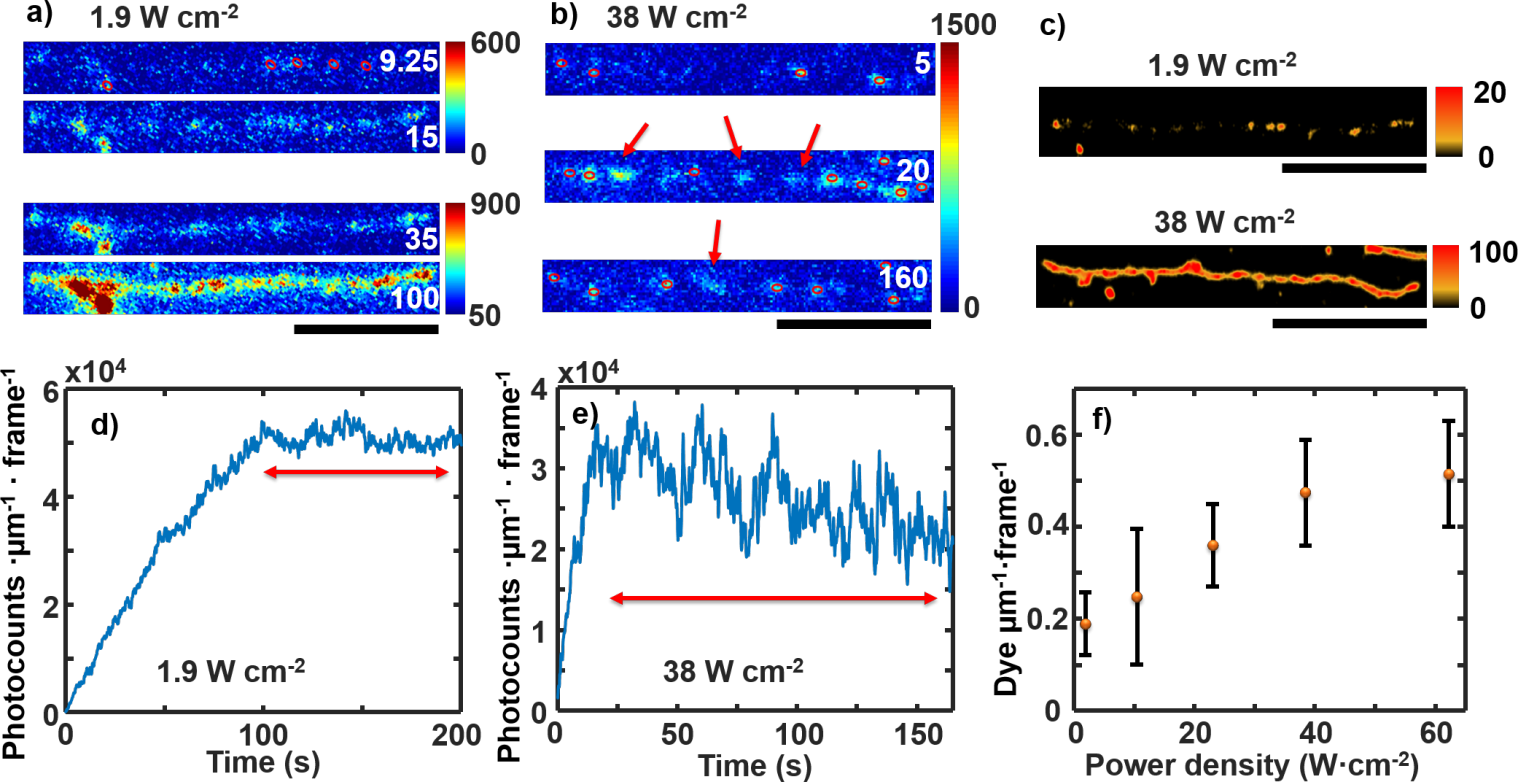
Corrected Figure 8 of the original publication.

