# Supplementary material for: Photobleaching of YOYO-1 in super-resolution single DNA fluorescence imaging
Source: Beilstein J Nanotechnol. 2017 Nov 2;8:2296–306. doi: 10.3762/bjnano.8.229 (PMC5687005; doi:10.3762/bjnano.8.229)
Supplement: File 1 — Laser spot size, DNA length, PSF, and photocount histograms. [file Beilstein_J_Nanotechnol-08-2296-s001.pdf]

Supporting Information

for

Photobleaching of YOYO-1 in super-resolution single DNA fluorescence imaging

Joseph R. Pyle and Jixin Chen*

Address: Department of Chemistry and Biochemistry, Nanoscale and Quantum Phenomena Institute, Ohio University, Athens, Ohio 45701, USA

Email: Jixin Chen - chenj@ohio.edu

* Corresponding author

Additional Experimental Information

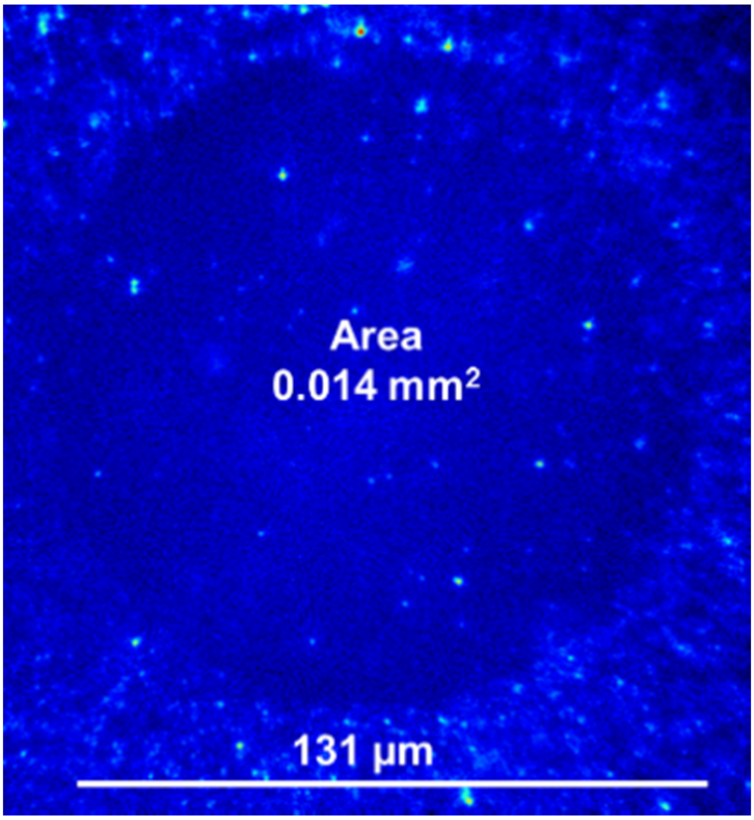

Figure S1: An image showing the laser spot size of the 100× objective in TIRF mode by viewing its bleaching pattern of fluorescent polymer dots in the 20× objective.

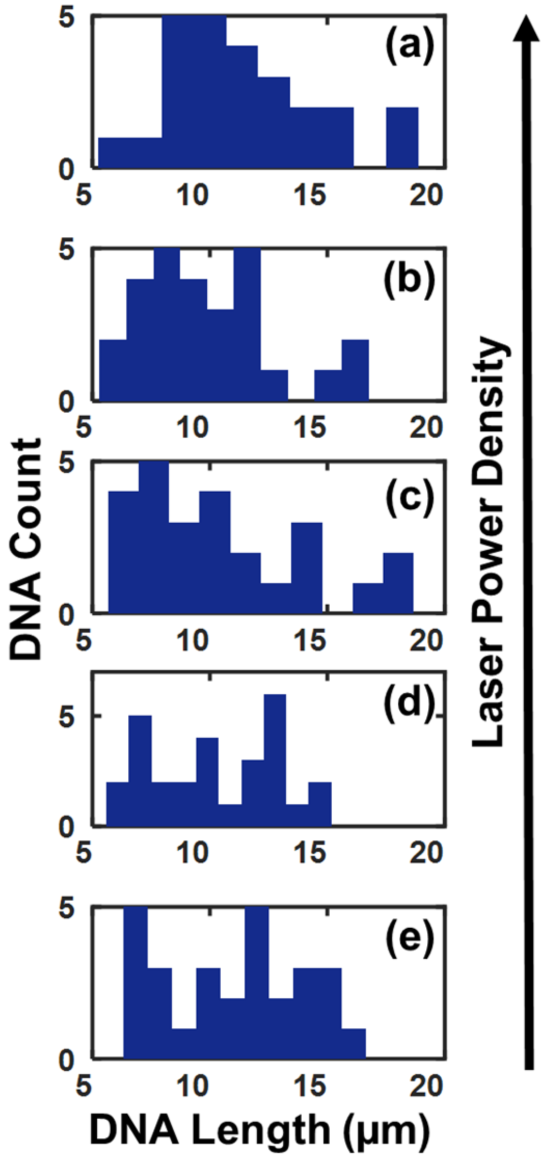

Figure S2: Histogram of the length of the DNA strands analyzed in the main text Figure 4 for power densities of 62 (a), 38 (b), 23 (c), 10 (d), and 1.9 (e) W/cm^2.

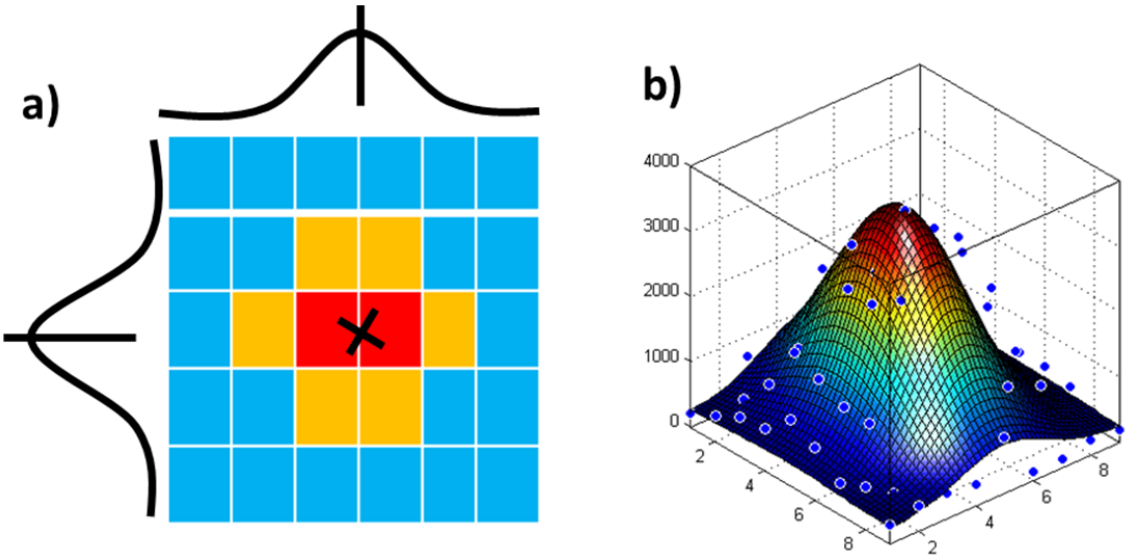

Figure S3: Scheme of a PSF on a CCD camera. The total photocounts of the PSF is the sum of the intensities of all pixels (a), which if fitted can be calculated as the volume of the 2D Gaussian peak (b).

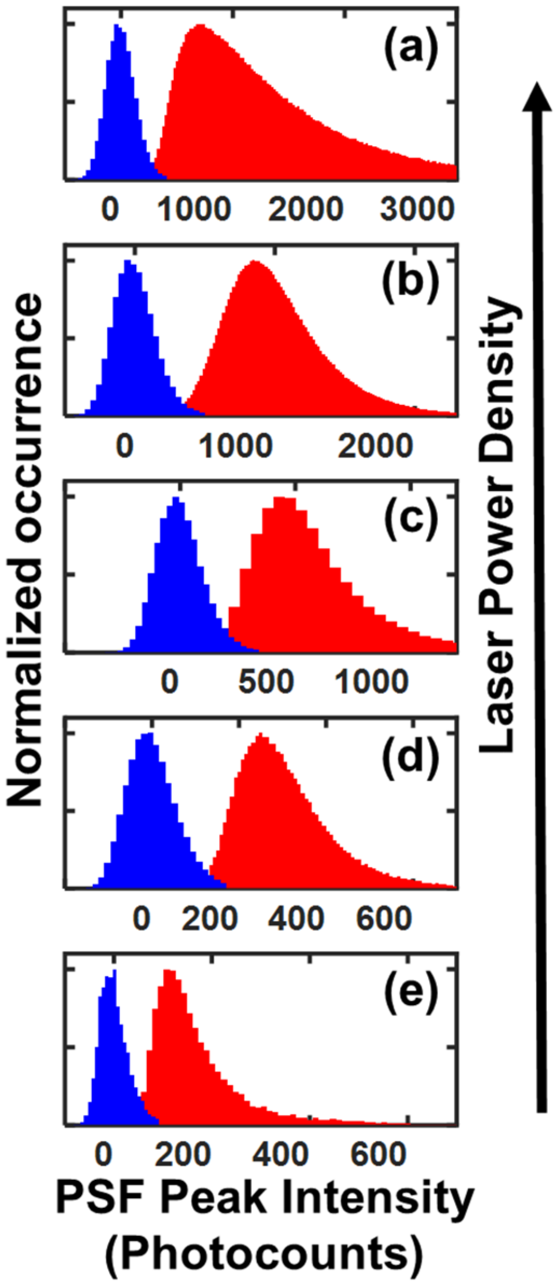

Figure S4: Background distributions and dye-peak-intensity distributions of sample videos at power densities of 62 (a), 38 (b), 23 (c), 10 (d), and 1.9 W/cm^2 (e). The peak intensity of the PSF of each dye analyzed for Figure 7 is shown in red. The background is shown in blue with its center set to zero.
